# Does physician experience influence the interpretability of focused echocardiography images performed by a pocket device?

**DOI:** 10.1186/s13049-015-0122-2

**Published:** 2015-07-07

**Authors:** Xavier Bobbia, Christophe Pradeilles, Pierre Géraud Claret, Camille Soullier, Patricia Wagner, Yann Bodin, Claire Roger, Guillaume Cayla, Laurent Muller, Jean Emmanuel de La Coussaye

**Affiliations:** Department of Anesthesiology, Emergency and Critical Care Medicine. Intensive Care Unit, Nimes University Hospital, place du Pr Debré, 30029 Nîmes, France; Department of Cardiology, Nimes University Hospital, place du Pr Debré, 30029 Nîmes, France

**Keywords:** Prehospital emergency care, Echocardiography, Efficiency

## Abstract

**Introduction:**

The use of focused cardiac ultrasound (FoCUS) in a prehospital setting is recommended. Pocket ultrasound devices (PUDs) appear to be well suited to prehospital FoCUS. The main aim of our study was to evaluate the interpretability of echocardiography performed in a prehospital setting using a PUD based on the experience of the emergency physician (EP).

**Methods:**

This was a monocentric prospective observational study. We defined experienced emergency physicians (EEPs) and novice emergency physicians (NEPs) as echocardiographers if they had performed 50 echocardiographies since their initial university training (theoretical training and at least 25 echocardiographies performed with a mentor). Each patient undergoing prehospital echocardiography with a PUD was included. Four diagnostic items based on FoCUS were analyzed: pericardial effusions (PE), right ventricular dilation (RVD), qualitative left ventricular function assessment (LVEF), and inferior vena cava compliance (IVCC). Two independent experts blindly evaluated the interpretability of each item by examining recorded video loops. If their opinions were divided, then a third expert concluded.

**Results:**

Fourteen EPs participated: eight (57 %) EEPs and six (43 %) NEPs. Eighty-five patients were included: 34 (40 %) had an echocardiography by an NEP and 51 (60 %) by an EEP. The mean number of interpretable items by echocardiography was three [1; 4]; one [0; 2.25] in the NEP group, four [3; 4] in EEP (p < .01). The patient position was also associated with interpretable items: supine three [2; 4], “45°” three [1; 4], sitting two [1; 4] (p = .02). In multivariate analysis, only EP experience was associated with the number of interpretable items (p = .02). Interpretability by NEPs and EEPs was: 56 % vs. 96 % for LVF, 29 % vs. 98 % for PE, 26 % vs. 92 % for RVD, and 21 % vs. 67 % for IVCC (p < .01 for all).

**Conclusion:**

FoCUS with PUD in prehospital conditions was possible for EEPs, It is difficult and the diagnostic yield is poor for NEPs.

## Background

Focused cardiac ultrasound (FoCUS) has been widely recommended in emergency medicine [1–3]. It has been demonstrated that FoCUS facilitates decision-making mainly in a binary (yes or no) fashion [1]. The American Society of Echocardiography (ASE) and the American College of Emergency Physicians (ACEP) define the elements of the FoCUS process [3]: 1. diagnosis and quantification of pericardial effusion (PE); 2. assessment of global cardiac systolic function by evaluation of qualitative left ventricular function (LVF); 3. identification of marked right ventricular dilation (RVD); 4. intravascular volume assessment, especially by assessment of inferior vena cava compliance (IVCC); 5. guidance of pericardiocentesis; 6. confirmation of venous pacing wire placement. Five ultrasound views are recommended [1]: parasternal short axis (PSA), parasternal long axis (PLA), apical four chambers (AFC), subcostal four chambers (SFC), and IVC view. Pocket size ultrasound devices (PUDs) are well adapted to emergency medicine, especially in out-of-hospital conditions. Such devices are less expensive and showed a good diagnostic accuracy compared with conventional machines [4–6]. Additionally, PUDs can favorably influence therapeutic decisions and/or patient orientation in prehospital conditions [7, 8]. Particularly, a favorable impact for echocardiography performed by PUDs has been shown for cardiac arrest [7, 9]. All prehospital patients with symptoms indicating echocardiography should theoretically benefit from faster and more accurate diagnoses. A recent study showed that prehospital echocardiography performed with a PUD was feasible in half of patients and required only a short physician training period [10]. However, the impact of emergency physician (EP) experience on FoCUS accuracy when using a PUD was not reported.

The primary aim of the present study was to compare interpretable items between experienced emergency physicians (EEPs) and non-experienced physicians (NEPs). The second aim was to determine if other variables were associated with the number of interpretable items.

## Materials and methods

### Materials

The local ethics committee of Nimes Teaching Hospital, France, approved the study (Interface Recherche Bioéthique No. 12/12-03). This monocentric prospective observational study was conducted from December, 2012 to February, 2013 on the mobile resuscitation ambulances of Nimes, France. This unit included 14 trained EPs. The university FoCUS training in emergency medicine involved theoretical teaching and at least 25 echocardiographies performed with supervision. All patients requiring a FoCUS exam in prehospital setting were included. Each patient was informed of the study verbally, as well as by letter, and had an opportunity to withdraw their data. The EP who performed echocardiography was also in charge of the patient’s clinical care. Echocardiography was performed using a PUD (Vscan™; GE Healthcare, Milwaukee, Wisconsin, USA).

### Aims

The main aim of the present study was to seek correlation between interpretable items (PE, RVF, RVD, and IVCC) by echocardiography and EP experience. The secondary aim was to search for other associated factors of interpretable items. Other aims were to evaluate the quality and interpretability of ultrasound views based on EP experience.

### Endpoints

The following data were recorded: age, gender, body mass index (BMI), primary indication of care, respiratory distress state, arterial pressure, shock state, Glasgow coma scale (GCS), mechanical ventilation, echocardiography exam location (home, street, care institution, ambulance, or helicopter), and patient position during examination (supine, 45°, or sitting).

Two groups of physicians were defined: EEP and NEP. The threshold for defining EEP or NEP was more or fewer than 50 echocardiographies already performed, respectively, after initial training [11].

To assess the quality and interpretability of ultrasound views, a five-point scale [12] was used: 1 = no image; 2 = poor and unusable image quality; 3 = usable image quality; 4 = good image quality; and 5 = perfect image quality. A value of three or more defined an interpretable image scan of sufficient quality that it could provide a diagnosis.

Two independent experts blindly evaluated the interpretable items by exams and the interpretability and image quality of the recorded echocardiogram video loops. The first expert was an echocardiography-referent cardiologist at our university hospital. This expert holds an echocardiography university diploma and is a teacher in echocardiography university courses for cardiologists. The second expert was an echocardiography-referent intensivist, who holds an echocardiography university diploma and is a teacher in echocardiography university courses for cardiologists, intensivists, and EPs. If their opinions were divided on the primary endpoint, a third expert made a conclusion (an EP teacher on emergency echocardiography). Experts had no information about the patient or clinical conditions. The video loops were recorded by the EPs and reviewed by the experts on a computer.

### Sample size

Our hypothesis was that the mean number of items achieved by NEPs and EEPs would be 1.5 and 2.5, respectively, with a common standard deviation of 1.5 and alpha and beta risk 0.05, so we had to include at least 26 patients per group. As a buffer, we planned to include 20 % more patients, or at least 32 in the smaller group.

### Statistical analysis

In all cases, data were examined before analysis to ensure that the assumptions of statistical models were satisfied using Shapiro–Wilk statistics. Data are expressed as mean and standard deviation (SD) or median (25th–75th percentile) depending on their distribution (Gaussian or not). For the comparisons between NEP and EEP, the Student’s t-test, Chi square, and Fisher exact tests were performed when appropriate. When a comparison was made to quantitative values of more than two groups with theoretical numbers less than five, subgroups were created. For the multivariate analysis, the dependence of the number of interpretable items (principal end point) on two or more other variables was evaluated by multiple linear regression analysis. The endpoints related to p < .2 in bivariate analysis were included in the multiple linear regression analysis. Bivariate correlations among variables were calculated to check for potential multicollinearity. Statistical analysis was performed using R Project (free software foundation, GNU general public license). All p values were two-tailed and p < .05 was considered significant. Agreement between the experts was evaluated using Cohen’s κ test.

## Results

Among the 85 included patients (each patient had one echocardiography evaluated), 34 (40 %) had an echocardiography performed by an NEP and 51 (60 %) by an EEP. Regarding demographics and care, only the patients’ positions during examination were significantly different between the NEP and EEP groups (Table 1).

**Table 1:** Characteristics of the general population and comparison between experienced and novice emergency physician. EEP: experienced emergency physician; NEP: novice emergency physician (almost 50 echocardiographies after initial training); Results expressed in mean (SD) or number (%); BMI: body mass index; GCS: Glasgow coma scale

Characteristics	Missed data	All patients N = 85	Echocardiography by NEP N = 34 (40 %)	Echocardiography by EEP N = 51 (60 %)	p value
Age (years)	7(8 %)	67 (18)	68 (18)	66 (17)	0.65
Women	5 (6 %)	34 (42 %)	14 (41 %)	20 (43 %)	0.98
BMI	6 (7 %)	25 (6)	25 (5)	26 (7)	0.84
Primary indication of care					
Chest pain		44 (52 %)	16 (47 %)	28 (55 %)	
Dyspnea	0	33 (39 %)	15 (44 %)	18 (35 %)	0.63
Syncope		4 (5 %)	1 (3 %)	3 (6 %)	
Cardiac arrest		3 (3 %)	2 (6 %)	1 (2 %)	
Thoracic trauma		1 (1 %)	0	1 (2 %)	
GCS	0	14 (3)	14 (3)	14 (3)	0.85
Systolic arterial pressure (mmHg)	1 (1%)	139 (33)	138 (32)	140 (28)	0.74
Shock state	0	3 (3 %)	2 (6 %)	1 (2 %)	NA
Respiratory distress state	0	20 (24 %)	7 (21 %)	13 (25 %)	0.6
Mechanical ventilation	0	7 (8 %)	4 (12 %)	3 (6 %)	NA
Echocardiography realization location					
Home	1 (1 %)	36 (43 %)	16 (47 %)	20 (40 %)	
Ambulance		36 (43 %)	11 (32 %)	25 (50 %)	0.24
Care institution		10 (12 %)	6 (18 %)	4 (8 %)	
Street		1 (1 %)	0	1 (2 %)	
Helicopter		1 (1 %)	1 (3 %)	0	
Patient position during examination					
Supine	0	30 (36 %)	7 (21 %)	23 (45 %)	0.01
45°		41 (48 %)	17 (50 %)	24 (47 %)	
Sitting		14 (16 %)	10 (29 %)	4 (8 %)	
Duration of echocardiography (s)	0	162 (97)	184 (111)	151 (90)	0.11

Fourteen EPs participated (experience in emergency medicine averaged six [2; 13] years ), eight (57 %) EEPs, and six (43 %) NEPs. The judgements of the experts in interpretability and image quality are shown in Table 2. The third expert was needed to assess the interpretability of echocardiography for 19 (22 %) LVF, 19 (22%) PE, 16 (19 %) RVD, and 21 (25 %) IVCC.

**Table 2:** Interpretability of items, views, and image quality by the two experts

	**Expert 1**	**Expert 2**	**Cohen kappa**
	**N = 85**	**N = 85**	
**Interpretable Goals**			
**Qualitative left ventricular function**			.4
Interpretable	62 (73 %)	56 (66 %)	
Normally	37 (60 %)	39 (69 %)	
Moderate*	16 (26 %)	15 (27 %)	
Severe**	9 (14 %)	2 (4 %)	
**Pericardial effusion**			.56
Interpretable	62 (73 %)	47 (55 %)	
No	56 (90 %)	41 (87 %)	
Yes	6 (10 %)	6 (13 %)	
**Right ventricular dilation**			.48
Interpretable	55 (65 %)	49 (58 %)	
No	45 (82 %)	45 (92 %)	
Moderate	8 (14 %)	2 (4 %)	
Severe	2 (4 %)	2 (4 %)	
**Inferior vena cava compliance**			.46
Interpretable	39 (46 %)	34 (41 %)	
0 %	8 (21 %)	9 (26 %)	
1 – 39 %	15 (38 %)	11 (31 %)	
40 – 99 %	11 (28 %)	5 (14 %)	
100 %	5 (13 %)	10 (29 %)	
**Image Quality**			
**Parasternal short axis**			
Interpretable	26 (31 %)	22 (26 %)	.83
Mean scale	2 ± 1	1.9 ± 0.9	.81
**Parasternal long axis**			
Interpretable	41 (48 %)	36 (42 %)	.6
Mean scale	2.3 ± 1	2.2 ± 1	.54
**Apical four chambers**			
Interpretable	47 (55 %)	52 (61 %)	.64
Mean scale	2.6 ± 1	2.5 ± 0.9	.51
**Subcostal four chambers**			
Interpretable	34 (40 %)	33 (39 %)	.69
Mean scale	2.6 ± 1.3	2.1 ± 1.1	.62
**Inferior vena cava view**			
Interpretable	41 (48 %)	36 (42 %)	.6
Mean scale	2.5 ± 1.3	2.2 ± 1	.47

Quality scale: 1 = no image, 2 = poor and unusable image quality, 3 = usable image quality, 4 = good image quality, and 5 = perfect image quality. Interpretable = 3 or more. *Moderate alteration (50 – 30 %), **Severe alteration (<30 %)

**Fig. 1:**
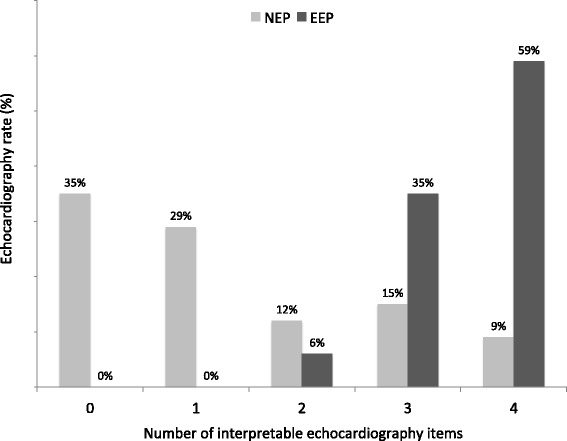
Number of interpretable echocardiography items for each exam according to physician experience. EEP: experienced emergency physician; NEP: novice emergency physician (almost 50 echocardiographies after initial training); “Echography rate” is the rate of examinations with 0, 1, 2, 3, or 4 interpretable items

**Fig. 2:**
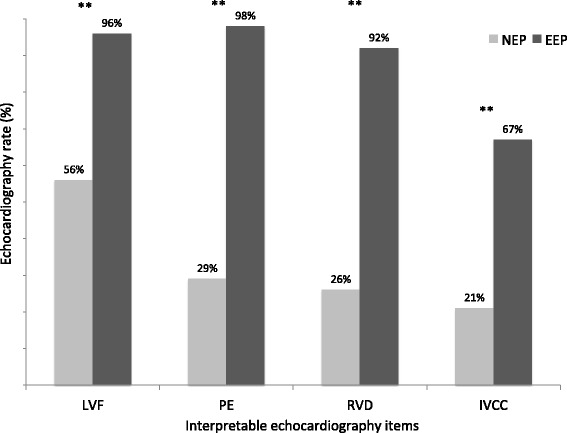
Interpretable echocardiography items according to physician experience. EEP: experienced emergency physician; NEP: novice emergency physician (almost 50 echocardiographies after initial training); “Echography rate” is the number of examinations in which the item is interpretable; LVF: qualitative left ventricular function; PE: pericardial effusion; RVD: right ventricular dilation; IVCC: inferior vena cava compliance; ** p < .05

The mean number of interpretable items by echocardiography was three [1;4]; one [0;2.25] in the NEP group, four [3; 4] in the EEP group (p < .01). The correlations between interpretable echocardiography items for each exam according to the EP’s experience are shown in Fig. 1. LVEF was interpretable in 68 (80 %) patients, PE in 60 (81%), RVD in 56 (66 %), and IVCC in 41 (48 %). Fig. 2 shows interpretability of each echocardiography item, according to the EP’s experience.

In bivariate analysis, only one other factor was associated with interpretable items: patient position during examination (supine three [2; 4], “45°” two [1; 4], sitting two [1; 4]; p = .02). In multivariate analysis, only EP experience in echocardiography (EEP or NEP) was associated with the number of interpretable items (p = .02).

## Discussion

### Main result

Our study shows that, by using a PUD, there is a significant difference in echocardiography items (four [3; 4] vs. one [0; 2.25], p < .01) between most-trained (>50 exams) and less-trained (<50 exams) operators. This result raises a concern on the widespread use of PUD without previous adequate training.

In our study, the good diagnostic performance observed with PUD in trained EPs is consistent with the literature. Pocket ultrasound devices are shown to be effective diagnostic tools in emergency, cardiology, and haemodialysis outpatients [6, 13–15]. Such devices are mainly adapted to some specific diagnoses, such as severe left/right ventricular failure, severe hypovolemia, or large pericardial effusion. Pocket ultrasound devices can be used by less-trained EPs after a specific learning program [13, 14]. In a recent study, Carrié et al. [16] showed that after 30 supervised and goal-oriented examinations, residents could adequately answer clinical questions covered by core applications of emergency ultrasound. Our results suggest that in prehospital clinical practice, a threshold of 50 examinations is preferable.

The first explanation for the present results is that the small screen size and poor prehospital examination conditions present difficulties for NEPs. Thus, it could be hypothesized that exams performed by an NEP take longer than those performed by an EEP. In the present study, EEPs performed good echocardiograms in 2.5 minutes. By contrast, NEPs performed bad exams in 3 minutes. Perhaps they need more time, but do not take it.

In the present report, there is a trend to a higher use of echocardiography in chest pain for EEPs than for NEPs. It could be argued that analyzing chest pain probably implies a more complex echocardiographic analysis than other diagnoses. Particularly, analyses of segmental wall motion abnormalities or aortic disease can be discouraging for novices who are not confident enough. There is also a trend to a lower utilization of echocardiography during acute dyspnoea. This could be explained by the fact that EEPs can also use other echo techniques for the diagnosis of dyspnea, especially the lung ultrasound, which is shown to have a high specificity and sensitivity in short-breathing patients [17, 18]. These two issues could lead to a bias. As the differences are not significant, we cannot make a definitive conclusion.

The learning curve of echocardiography is probably slower for PUD than for conventional ultrasound machines. Recent international recommendations state that “*only appropriately trained practitioners should practice FoCUS*” and that exact specifications of skills and appropriate number of supervised and unsupervised scans depend on the specialty of the EP [1]. It can be hypothesized that echocardiography training in the prehospital setting is longer than for conventional echocardiography. We hypothesize that it is learning echocardiography with a PUD that requires more experience. Studies with PUD used by experienced practitioners showed good results: [4, 6]. When training was shorter, results were poorer [10]. These points can be an obstacle for the widespread use of this application because a minority of EPs are trained [19–21]. However, the proportion of emergency physicians experienced in echocardiography is increasing and new generations will likely have better technological skills and will be more rapidly able to use PUD [22].

### Originality of the study

In previous studies related to PUD, all examinations were performed by one or two EPs and/or with standardized training [6, 10, 23, 24]. The first originality of the present study is that it evaluates a larger EP population compared with previous studies. This probably helps to generalize the results because the training of EPs in a unit is often heterogeneous.

The second originality of the present study is that the primary endpoint was interpretable echocardiography items. We hypothesize that interpretable items better reflect the clinical usefulness of PUD in the prehospital setting than image quality itself. The most difficult item to record was inferior vena cava compliance (less than one in two). It was earlier reported as a simple item to record and analyze under good examination conditions [13]. One hypothesis for this result is that patients were often not supine, dyspnoeic, or had chest pain. Moreover, only one view (subcostal) shows the inferior vena cava. Therefore, when that view is not possible, the item cannot be analyzed. This reinforces the idea that inferior vena cava imagery is not easy and should be interpreted with caution, especially in spontaneously breathing patients [25]. Other items were easy for EEPs. However, only the estimation of LVEF was possible in more than one in two exams performed by NEPs. Unlike the IVCC, this result is probably because it can be estimated in all views.

### Study limitations

The present study has limitations. The first limitation is that it is a monocentric study, affecting the generalizability of the results. The inclusion criteria were set to include each patient requiring echocardiography in the prehospital period. Therefore, patient inclusion was based on the EP’s evaluation of the need for a prehospital FoCUS exam. Video loops of echocardiograms were used to make objective assessments. Although common [10, 12], this methodology probably underestimates interpretability. This study supports the conclusion only for interpretability. However, it is likely that diagnostic performance is correlated. Additionally, agreement between the two experts for endpoints was moderate and a third expert was necessary in less than one in four. Finally, the quality of experts could be questioned. The correlation between the experts for interpretable goals was low, whereas the two experts were in accordance for image quality (Table 2). One explanation for such a discrepancy could be that the recorded loops were very short (just a few seconds). This limited duration may decrease the diagnostic accuracy and affect inter-observer variability. To improve this issue, a third expert was mandated and had to intervene in 19 – 25 % of cases.

## Conclusion

The present study compared, for the first time, the interpretability of images acquired by highly and less-trained echocardiography emergency physicians using a pocket ultrasound device in a prehospital setting. Less experience by the EP significantly reduces the interpretability of focus echocardiography performed under these conditions. It is likely, therefore, that physician experience affects diagnostic performance. The training of echocardiography physicians appears to be a key issue for using pocket ultrasound devices in prehospital settings.

## Abbreviations

FoCUS: Focused cardiac ultrasound
ASE: American Society of Echocardiography
ACEP: American College of Emergency Physicians
PE: Pericardial effusion
LVF: Left ventricular function
RVD: right ventricular dilation
IVCC: Inferior vena cava compliance
PSA: Parasternal short axis
PLA: Parasternal long axis
AFC: Apical four chambers
SFC: Subcostal four chambers
PUD: Pocket size ultrasound device
EEP: Experienced emergency physician
NEP: Non experienced emergency physician
EP: Emergency physician
BMI: Body mass index
GCS: Glasgow coma scale
SD: Standard deviation
